# Mechanism of Action of circRNA/miRNA Network in DLBCL

**DOI:** 10.3390/ncrna11020022

**Published:** 2025-03-04

**Authors:** Elena Golovina, Cory Eaton, Virginia Cox, Jozef Andel, Karina Savvulidi Vargova

**Affiliations:** 1 First Faculty of Medicine, Institute of Pathological Physiology, Charles University, 12108 Prague, Czech Republic; egolo@lf1.cuni.cz (E.G.); 26754232@cuni.cz (C.E.);; 2 Faculty of Science, Molecular Biology and Genetics of Eukaryotes, Charles University, 12800 Prague, Czech Republic

**Keywords:** circRNA, miRNA, B-cells, lymphoma, gene expression

## Abstract

Circular RNAs (circRNAs) make up approximately 10% of the human transcriptome. CircRNAs belong to the broad group of non-coding RNAs and characteristically are formed by backsplicing into a stable circular loop. Their main role is to regulate transcription through the inhibition of miRNAs’ expression, termed miRNA sponging. CircRNAs promote tumorigenesis/lymphomagenesis by competitively binding to miRNAs at miRNA binding sites. In diffuse large B-cell lymphoma (DLBCL), several circRNAs have been identified and their expression is related to both progression and response to therapy. DLBCL is the most prevalent and aggressive subtype of B-cell lymphomas and accounts for about 25% to 30% of all non-Hodgkin lymphomas. DLBCL displays great heterogeneity concerning histopathology, biology, and genetics. Patients who have relapsed or have refractory disease after first-line therapy have a very poor prognosis, demonstrating an important unmet need for new treatment options. As more circRNAs are identified in the future, we will better understand their biological roles and potential use in treating cancer, including DLBCL. For example, circAmotl1 promotes nuclear translocation of *MYC* and upregulation of translational targets of MYC, thus enhancing lymphomagenesis. Another example is circAPC, which is significantly downregulated in DLBCL and correlates with disease aggressiveness and poor prognosis. CircAPC increases expression of the host gene adenomatous polyposis coli (APC), and in doing so inactivates the canonical Wnt/β-catenin signaling and restrains DLBCL growth. MiRNAs belong to the non-coding regulatory molecules that significantly contribute to lymphomagenesis through their target mRNAs. In DLBCL, among the highly expressed miRNAs, are miR-155-5p and miR-21-5p, which regulate NF-ĸB and PI3K/AKT signaling pathways. The aim of this review is to describe the function and mechanism of regulation of circRNAs on miRNAs’ expression in DLBCL. This will help us to better understand the regulatory network of circRNA/miRNA/mRNA, and to propose novel therapeutic targets to treat DLBCL.

## 1. Etiology, Biology of DLBCL, and Current Therapeutic Options

Diffuse large B-cell lymphoma (DLBCL) represents the most aggressive and the most common lymphoma subtype in western countries and encompasses more than 30% of all non-Hodgkin’s lymphomas (NHL) in adults [1]. Approximately 40% of patients with DLBCL develop refractory or relapse DLBCL, which is the ultimate cause of patients’ death [2]. The etiology of most DLBCL cases is still unknown; however, we can distinguish cases as arising (1) de novo (as primary), or (2) arising from progressive or transformed (as secondary) less aggressive lymphomas (e.g., chronic lymphocytic leukemia/small lymphocytic lymphoma, follicular lymphoma, marginal zone lymphoma, or nodular lymphocyte predominant Hodgkin lymphoma) [3]. Among the main predisposing factors are genetic aberrations, status of immune system, hereditary diseases, and chemicals/drugs [3,4]. There is considerable involvement of Epstein–Barr virus (EBV) in lymphoma development, reviewed in [5]. The oncogenic potential of EBV consists of the reactivation of its latent form (latency program III), overexpression of *EBNA2* gene. *EBNA2* inhibits expression of miR-34a and this downregulation unblocks and stimulates expression of the *PD-L1* (Programmed death ligand 1) gene that results in the lower immunogenicity of B-cells [6]. Authors tested this relationship by temporal overexpression of miR-34a (by miRNA mimics) in U2932 EBNA2 cells and detected low expression of *PD-L1* [6]. Another latent protein (from latency program III), LMP1 (latent membrane protein 1), showed its importance in the lymphomagenesis (demonstrated in mice models) by interaction with Ebf1 and Rel genes [7]. Patients with DLBCL show high clinicopathological heterogeneity regarding histomorphology, immunophenotype, biological behavior, and molecular genetics. In 1993, the International Prognostic Index (IPI) was created to determine overall survival for DLBCL patients based on the patients’ age, performance status, and the extent and location of disease, and thus serves as an indicator of clinical prognosis. Based on the IPI score, we are able to divide DLBCL patients into two groups; (1) low IPI score (0–2), associated with better overall survival (OS), and (2) high IPI score (3–5), associated with worse OS [8]. There are also such other IPI scores as Original IPI, Revised IPI, NCCN IPI, Age-adjusted IPI, Stage-adjusted IPI, Cell of origin using GEP, Hans and Tally algorithms, Lymph2Cx platform, and others which are described in more details in [9]. Efforts have recently been made to improve the prognostic tools by applying knowledge from different prognostic models [10] and from gene analysis [11]. Overall, the genesis of lymphomas are due to typical chromosomal rearrangements, most frequently in the oncogenes *MYC*, *BCL*2, or *BCL6* [12]. In approximately 10% of DLBCL patients, there are detectable *MYC* rearrangements associated with worse prognosis and a lower OS [13]. Thus, recent studies have investigated the role of the abovementioned chromosomal aberrations as valuable biomarkers for DLBCL prognostication.

Based on the cell of origin (COO) and genomic-scale gene expression profiling (DNA microarray) by Alizadeh AA et al. (2000) [11], DLBCL is classified into three subtypes. First is the activated B-cell-like (ABC) subtype that is derived from cells of GC exit or post GC origin, with either germinal center-exit or early plasmablastic phenotype. ABC-DLBCL is characterized by dependence on BCR signaling and NFκB activities, is negative for most GC markers, and expresses *IRF4*/*MUM1* [14]. This subtype is enriched with BCR pathway mutations, such as in *MYD88* (mostly p.L265P), CD79B, and *PIM1*, as well as genetic changes that block the B-cell differentiation program, such as *BCL6* rearrangements and *PRDM1*/*BLIMP1* mutation/deletion [15]. Second, the germinal center B-cell-like (GCB) DLBCL subtype displays a gene expression profile (GEP) related to a GC cell of origin, and is defined by a IGH::BCL2 fusion due to a translocation, t(14;18)(q32;q21), and mutations of genes important for GC development, GC dark zone and light zone transitions and microenvironmental interactions, such as *EZH2*, *GNA13*, *MEF2B*, *KMT2D*, *TNFRSF14*, *B2M*, and *CREBBP* [15]. GCB-DLBCL is further characterized by CD10 expression and *BCL2* rearrangement. The third subtype represents unclassified DLBCL subtype or NOS (not otherwise specified) which contains approximately 10–15% of all DLBCL cases [11]. GCB-DLBCL shows significantly better OS with a five-year OS of 76% in comparison to ABC-DLBCL, with only 16% OS [11]. Recently, researchers performed whole-genome sequencing of GCB- and ABC-DLBCL patient samples to better understand the genetic complexity of DLBCL subgroups [16].

**Figure 1:**
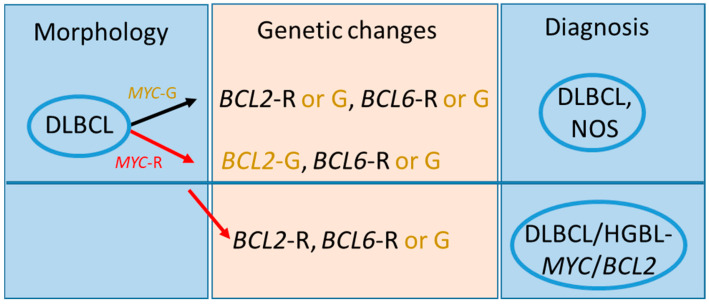
Scheme of classification of aggressive B-cell lymphomas in WHO-HAEM5 in the light of *MYC*, *BCL2*, and *BCL*6 rearrangement. R means rearrangement and G means germline configuration. The classification based on morphology should be confirmed by genetics to obtain correct diagnosis (modified and adapted from [17]).

Based on the fifth WHO-HAEM5, we now distinguish entities as “large B-cell lymphomas”, formerly DLBCL, HGBL, NOS, and others (for details see Figure 1) [17]. Recently, the International Consensus Classification (ICC) of mature lymphoid neoplasms points toward the importance of novel molecular technologies, mainly whole-genome sequencing single cell analysis, for more precise diagnoses of lymphoid malignancies [18]. We recognize several chromosomal rearrangements in the genome of the large B-cell lymphomas, among the most frequent are genes *MYC* and *BCL2* and/or *BCL6*. Rearrangements of genes *MYC* and *BCL2* and/or *BCL6* was formerly termed “double-hit” or “triple-hit” (DH/TH) and are detected in 2–8% of all DLBCL cases [13,19], whereas now the fifth WHO-HAEM5 classification has renamed it as “diffuse large B-cell lymphoma/high-grade B-cell lymphoma with *MYC* and *BCL2* rearrangements” (DLBCL/HGBL-*MYC*/*BCL2*) [17]. This homogenous group of lymphomas have an exclusive GC gene expression profile, pathogenically similar to FL (follicular lymphoma) and molecularly resembling GC-like DLBCL subtype [20].

**Figure 2:**
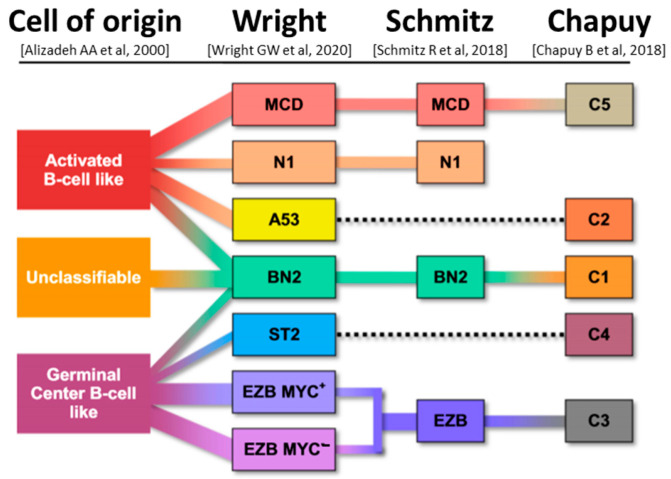
Molecular classification systems of diffuse large B-cell lymphoma according to gene expression profiling and the potential relationship between the molecular entities and the cell of origin groups. From left to right, first classification is based on the cell of origin and distinguishes: ABC, unclassifiable and GCB subtypes of DLBCL. Second, based on the genetic analysis called “LymphGen”, there are seven distinguished subtypes of DLBCL: MCD, N1, A53, BN2, ST2, EZB MYC^+^, and EZB MYC^-^. Third classification, also based on genetic analysis, distinguishes only four subtypes of DLBCL: MCD, BN2, N1, and EZB. The last, and current classification, C1–C5 represents clusters with different genetic aberrations and outcomes (adapted, modified from “Reproduced with permission from (Vodicka Prokop), (Onco Targets Ther); published by (Taylor & Francis), (2022).” [22]).

In contrast, lymphomas with dual *MYC* and *BCL6* rearrangements represent a more diverse group and have variable gene expression profiles and mutational spectra, making them markedly different from DLBCL/HGBL-MYC/BCL2 [21]. Therefore, in the 5th WHO classification, these lymphoma cases have been excluded from the DLBCL/HGBL-*MYC/BCL2* grouping and are now reclassified as a subtype of DLBCL, NOS, or HGBL, NOS according their cytomorphology and followed by genetic aberrations (Figure 1). Today, in the “sequencing” era, the classic morphologic evaluation of DLBCL is losing its significance and is slowly being pushed out by novel classification and prognostic systems based on molecular analysis. The molecular classification shows divergent groups of large B-cell lymphomas (Figure 2).

**Figure 3:**
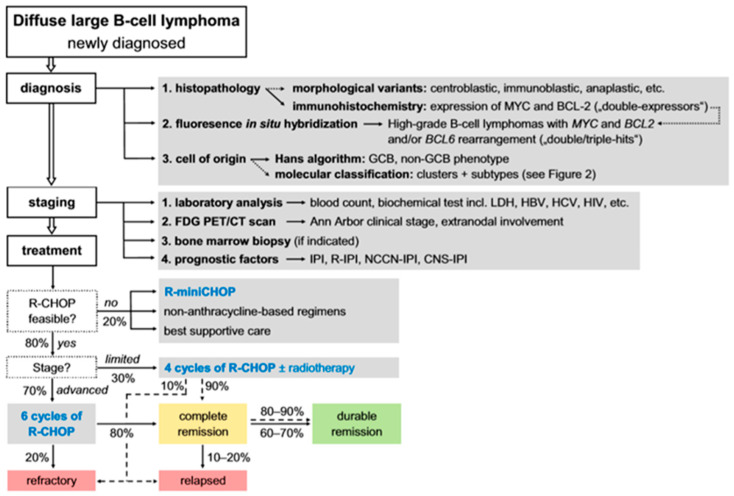
Management, scheme, and cure rates of patients with newly diagnosed DLBCL. Diagnosis of new DLBCL is based on histopathology, FISH method, and cell of origin. Staging of DLBCL is determined by laboratory analysis, FDG PET/CT scan, bone marrow biopsy, and prognostic factors. Front line treatment represents R-CHOP. Abbreviations: CNS = Central Nervous system; GCB = Germinal Center B-cell like; HBV = Hepatitis B virus; HCV = Hepatitis C virus; HIV = Human Immunodeficiency virus; IPI = International Prognostic Index; LDH = Lactate dehydrogenase; NCCN-IPI = National Comprehensive International Prognostic Index; R-IPI = Revised International Prognostic Index; R-CHOP = Rituximab (R) in combination with cyclophosphamide, doxorubicin, vincristine, and prednisone (adapted, modified from “Reproduced with permission from (Vodicka Prokop), (Onco Targets Ther); published by (Taylor & Francis), (2022).” [22]).

Fortunately, DLBCL (now LBCL) is now potentially curable with an overall 60–70% chance of being cured with the current first-line immunochemotherapy of rituximab (R) in combination with cyclophosphamide, doxorubicin, vincristine, and prednisone (R-CHOP) [22]. Despite this, 30–40% of the patients are either refractory to first-line treatment (i.e., no response to or with primary progression after treatment) or experience relapse (R/R). The detailed therapeutic management of newly diagnosed DLBCL is described in Figure 3. R-CHOP still remains the standard therapy for untreated DLBCL, with the number of cycles and the use of radiation therapy depending on the stage and tumor bulk [22].

**Figure 4:**
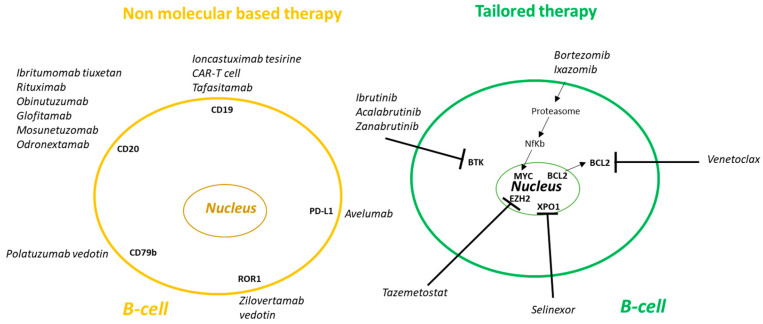
Therapeutic approaches in patients with newly diagnosed diffuse large B-cell lymphoma. There are two main therapeutic approaches: (1) Non molecular biology-based, includes Antibody-drug conjugates, Radioimmunotherapy, Monoclonal antibodies, CAR T-cell, Bispecific antibodies. (2) Tailored therapy—by targeting BCR/NF-kB pathway, BCL-2, XPO-1, EZH2, and Proteasome inhibitors. Abbreviation: CAR = Chimeric Antigen Receptor, BTK = Bruton tyrosine kinase (adapted, modified from “Reproduced with permission from (Vodicka Prokop), (Onco Targets Ther); published by (Taylor & Francis), (2022).” [22]).

There are multiple treatment modalities for DLBCL patients, including molecular-based (monoclonal Abs, Radioimmunotherapy, Bispecific Ab, Checkpoint inhibitors, Immunomodulatory drugs) and tailored therapy [22,23]. Figure 4 shows an overview of the current therapy options in DLBCL.

In brief, DLBCL is an aggressive and very heterogeneous malignancy that is caused by several chromosomal aberrations. Some cases of DLBCL are uncurable, and rapid development of molecular methods nowadays provide a vast number of therapy options. However, there is still a gap in the therapy for refractory cases and for determining prognosis.

## 2. MiRNAs in General and in DLBCL

**Figure 5:**
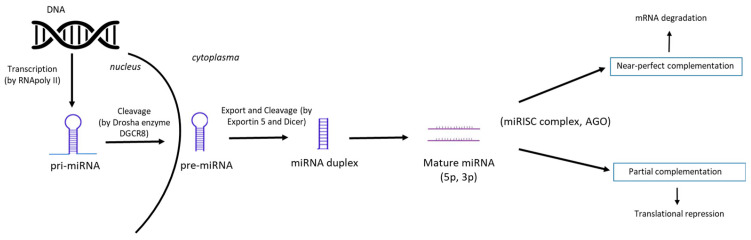
Scheme of miRNA biogenesis and mechanism of action. The first step is the formation of the primary transcript—pri-miRNA from DNA. After cleavage by the microprocessor complex, Drosha enzyme and DiGeorge Syndrome Critical Region 8 (DGCR8), the precursor pre-miRNA is formed. Then, the pre-miRNA is transported from the nucleus into the cytoplasm by Exportin 5 (by RanGTP-dependent manner). The mature miRNA duplex is processed (cleaved by Dicer) and 5p or 3p strands of the mature miRNA duplex are further loaded into the Argonaute (AGO) family of proteins to form a miRNA-induced silencing complex (miRISC). Finally, based on the complementarity with target mRNA, miRISC could degrade mRNA or result in translational repression, in case of partial complementarity (modified, adapted from [32]).

It has been 30 years since the first microRNA (miRNA, lin-4) was discovered in the roundworm *Caenorhabditis elegans* [24,25]. Around 20 years ago, miRNAs were identified as a large class of small non-coding RNAs among higher eukaryotes [26,27,28]. This initiated an in-depth investigation into their regulatory role that ultimately resulted in a shift in the paradigm of how information is transferred from DNA to RNA. During the next decade, researchers all around the world uncovered conserved miRNA loci within the genome, molecular factors involved in miRNA biogenesis and function, and multiple regulatory mechanisms that act through its targets [29]. MiRNAs act as negative regulators of gene expression at the post-transcriptional level by binding to complementary messenger RNA (mRNA) which will be either destroyed or preserved and translated later (reviewed in [30]). Figure 5 depicts the miRNA biogenesis and mechanism of their action. The importance of miRNAs is underlined by the fact that they regulate the development of human cells in a dose-dependent fashion [31].

Interestingly, the first miRNAs described in human leukemia, specifically chronic lymphocytic leukemia (CLL), were miR-15 and miR-16 [33]. The authors discovered that these two miRNAs directly target (inhibit) *BCL2* mRNA leading to cell apoptosis, which highlights the therapeutic potential of miRNAs [33].

It is known that expression of miRNAs is associated with DLBCL pathogenesis and progression. As described in one of the first studies, miR-15a, miR-16-1, miR-21, miR-29c, and miR-155 were significantly upregulated in the serum of DLBCL patients when compared to the samples from healthy donors [34]. A review by Alsaadi et al. (2021) summarizes the role of miRNAs in prognosis/diagnosis (e.g., miR-34a, miR-27b, miR-21, miR-22), cell cycle (e.g., miR-181, miR-195, miR-26a, miR-101), and the chemoresistance (e.g., miR-497, miR-199a miR-130a, miR-125b) of DLBCL [35].

A recent study based on small-RNA sequencing from DLBCL (de novo) serum samples revealed novel miRNAs (miR-200c-3p, miR-421, and miR-324-5p) which were associated with disease progression and response to therapy [36].

A systematic review (using tissue specimens) described that both miR-155-5p and miR-221-3p were highly upregulated in the ABC subgroup of DLBCL. These miRNAs repress PIK3R1 and thus activate the PI3K/AKT signaling pathway [37]. A comprehensive review of miR-155-5p and its use as a predictive biomarker for DLBCL highlights its significant role in DLBCL pathogenesis and progression [38]. Another recent study identified a positive correlation between disease prognosis/progression and the expression of miR-125b and miR-155 in tissue specimens of nonGC DLBCL patients [39]. Moreover, they found that *TP53* and *MYC* correlate with these miRNAs and could be used as valuable markers for DLBCL prognosis, but more data needs to be acquired from a larger patient cohort [39]. A miRNA sequencing (NGS) study of FFPE samples from DLBCL patients (ABC, GC) confirmed all the above-mentioned miRNAs’ relationship to the prognosis of DLBCL. In addition, it identified a difference between miRNA expression in GC vs. ABC subtype; miR-129-2-3p, miR-4464, miR-3150b-3p, miR-138-5p, and miR-129-5p were upregulated in GC DLBCL patients, and miR-511-5p, miR-205-5p, miR-3652 were upregulated in nonGC DLBCL subtype. Furthermore, the authors investigated the mechanisms by which the deregulated miRNAs contribute to DLBCL pathogenesis, demonstrating specifically that miR-182-5p plays a key role in the PI3K/AKT pathway by regulating *PTEN* upstream, and *FOXO1* downstream [40]. In another study, researchers concluded that miR-146a is associated with a good prognosis of DLBCL (de novo) when its expression level significantly increased in patients’ serum after chemotherapy (CHOP, R-CHOP) [41]. Thus, overexpression of miR-146a can lead to improved outcomes in DLBCL patients by precluding cell proliferation, promoting cell apoptosis, and accelerating the chemosensitivity of DLBCL cells to the chemotherapy. Table 1 summarizes miRNAs detected in DLBCL, their role/function, origin, and biological pathway of action.

In brief, miRNAs represent valuable and reliable markers of prognosis/progression in DLBCL. Although there are many studies that have tried to identify specific miRNAs that are significantly changed in DLBCL samples, there continues to be great heterogeneity in the list of deregulated miRNAs in DLBCL. Therefore, further large patient cohort studies are needed to obtain a more stringent list of miRNA biomarkers.

## 3. CircRNAs in General

**Figure 6:**
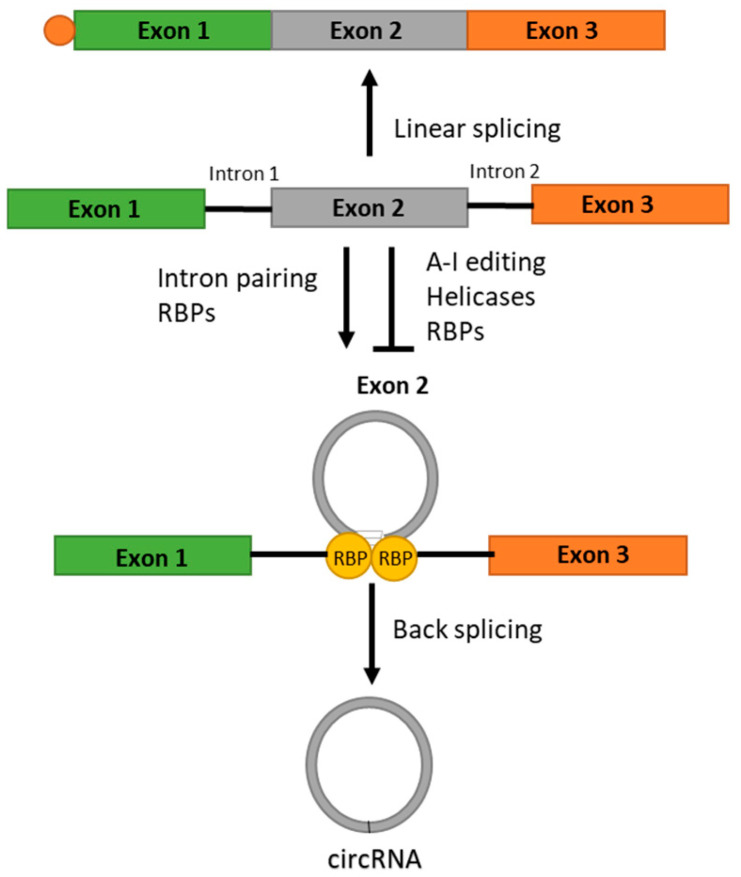
Linear splicing and backsplicing, biogenesis of circRNAs—Schematic of linear splicing (top) and backsplicing (bottom). Linear splicing is common for mRNAs of coding genes. CircRNAs are generated via backsplicing; from exons of coding genes, concretely the upstream 3′ splice site of exon is joined to a downstream 5′ splice site that results in the junction of the 3′ end of an exon with the 5′ end of the same or upstream exon(s). Here, exon 2 gives rise to circRNA. Intron pairings and some RBPs (RNA-binding proteins) can enhance back splicing. By contrast, in A-I editing (adenosine-to-inosine), some helicases and other RBPs can inhibit backsplicing and hence inhibit circRNA production (modified, adapted from [56]).

The earliest reported presence of circular RNAs (circRNA) was in 1976, where an electron microscopy-based study first detected circRNA in plant viroids [39] and later circRNAs were detected in humans, mice, rats, fungi and other organisms [42,43,44,45,46]. Only a small portion of circRNAs have been identified in the last 30 years because of few reliable high-throughput detection methods [42,43,47,48,49,50,51]. Initially, circRNAs did not receive much attention due to their structural specificity, unknown functions and their low abundance (in comparison to their linear counterparts), thus they were first considered as aberrant byproducts of splicing [49]. The difference between circRNAs and their linear counterparts is in the backsplicing junction [52]. CircRNAs are endogenous, closed molecules that belong to the group of non-coding RNAs. They are without a 5′ cap and a 3′ poly-(A) tail, giving them the stability to exist longer in a cellular environment compared to other RNA molecules. Formed through a process known as backsplicing, which changes the order of exons in comparison to linear splicing, the leading hypothesis for the formation of circRNAs is based on the canonical RNA splicing system as follows (Figure 6). Backsplicing is the process in which pre-mRNA splicing occurs in a reversed order so that the upstream 3′ splice site is combined with the downstream 5′ splice site [53]. After back splicing, circRNAs retain introns that can associate with U1 small nuclear ribonucleoproteins (U1snRNPs), which then enhance transcription through the recruitment of Pol II at promoter sites [54]. CircRNAs can be classified based on their structural components and biogenesis in three ways that each have their own respective function and cellular localization. Such forms include exonic circRNAs, circular intronic RNAs, and exon-intron circRNAs, with the latter being the most common, and are typically found in the nucleus [55]. It is known that circRNAs are transcribed by RNA polymerase II and are then created via the spliceosome that is composed of heterogeneous nuclear riboproteins (hnRNPs) and serine- and arginine-rich proteins [56].

CircRNAs are found to be abundant amongst specific tissues, making up approximately 20% of the genes that are expressed in the brain and 9% of those expressed in the heart [57]. Particularly, this is found to be true within cells that have a lower capacity to proliferate, such as neurons and cardiomyocytes relative to tissues, such as the liver, that have a higher capacity to replicate [58]. As neurons and cardiomyocytes do not readily replicate, there is evidence supporting a natural age-related accumulation of circRNAs in these tissues [59,60]. However, this may also be explained by their intrinsic stability. Moreover, in a comprehensive analysis completed by Nicolet BP et al. 2018 [61], circRNAs were found to be widespread in human hematopoietic progenitor cells in addition to differentiated lymphoid and myeloid cells. In the study, the authors concluded that circRNAs’ expression in hematopoiesis is altered during differentiation and that expression is cell-type specific [61]. According to the implications of this study, more research will need to be performed to elucidate how circRNA expression can influence the regulatory processes, in addition to how to distinguish healthy from diseased hematopoietic cells.

Given that circRNAs are without free ends, this confers a resistance to RNase and other exonucleases, thus increasing their half-lives [62,63]. It has been stated that this unique feature may allow circRNAs to serve as biomarkers for various diseases, particularly cancer. While circRNAs may be degraded by other RNases (including RNase A, RNase T1, and RNase T2), there is evidence that protein-mediated microRNAs (miRNAs) may also serve as regulators of circRNAs’ longevity [64]. Hansen TB et al. (2011) [65] determined that a circular antisense transcript of the Cerebellar Degeneration-Related protein 1 locus (CDR1) is cleaved through the actions of the miR-671.

Although the primary function of circRNAs is to regulate transcription through the inhibition of miRNAs acting as “miRNA sponges,” several other functions of circRNAs exist. It is known that RNA-binding proteins have significant interactions with RNA molecules through various metabolic processes, such as the maturation and transportation of RNA molecules [66].

**Figure 7:**
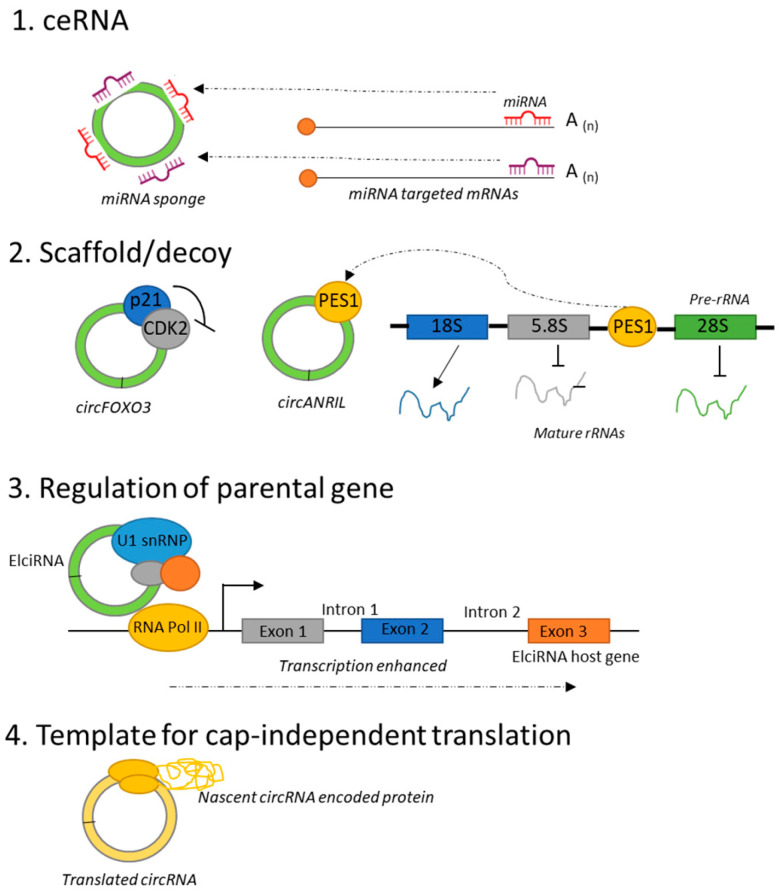
Molecular general functions of circRNAs. (1) circRNAs can act as competing endogenous RNAs (ceRNAs) by sequestering and/or stabilizing miRNAs. This mechanism of acting is also called miRNA sponge—inhibition of miRNA function by occupying binding sites (by competition) of miRNAs by circRNAs. (2) circRNAs can function as scaffolds or decoys for RBPs; the examples of circFOXO3 and circANRIL are shown here. CircFOXO3 inhibits cell cycle progression by binding to or blocking CDK2. Similarly, circANRIL blocks protein PES1 that suppresses ribosome biogenesis. (3) circRNAs can also regulate the transcription of their parental (linear counterpart) gene, as occurs in the case of elciRNA, which controls the expression of its mRNA counterpart through the binding of small nuclear ribonucleoproteins (snRNPs) and the modulation of RNA polymerase II activity. (4) Some circRNAs can act as a template for cap-independent translation (modified, adapted from [52]).

Studies have demonstrated the breadth of which circRNAs and proteins interact, and so they influence numerous biological functions such as gene transcription and cell cycle regulation. For instance, Du X et al. 2017 [60] determined that circFOXO3 is able to modulate the ubiquitination function of *MDM2*, thus preventing degradation of p53, and so is able to induce apoptosis in breast cancer cells. In a review completed by Huang A et al. (2020) [63], they list out the numerous functions of circRNA-protein interactions (interested readers should consult the review for more details). Additional functions of circRNA-protein interactions include the ability of circRNAs to function as protein sponges, protein decoys, protein scaffolds, and to recruit proteins (Figure 7).

As circRNAs are becoming more of an interest, new associations between the role of circRNAs and various types of cancer are being discovered. In a review by D van Strijp et al. 2017 [67], circRNA expression was stated to have several roles either as oncogenes or tumor suppressors in almost all cancer types. For instance, in a study by Han D et al. (2017) [68], circMTO1 (mitochondrial translation optimization 1 homolog) was found to be downregulated, which suppressed hepatocellular carcinoma progression by acting as a sponge to the oncogene miR-9 and thus increasing expression of p21.

### 3.1. CircRNAs During B-Cell Development

There are several studies that highlight the important regulatory role of non-coding RNAs (miRNAs) and other regulatory RNAs, such as circRNAs in the development of B-cells; nevertheless, little is known about the circRNA profiles and ceRNA (competing endogenous) networks during B-cell development. A sequential study (whole transcriptome) on mouse bone marrow cells revealed dynamic expression patterns of circRNAs: 35 upregulated circRNAs and 64 downregulated circRNAs during the pro-B to pre-B-cell stage, 71 upregulated circRNAs and 75 downregulated circRNAs during the pre-B to immature B-cell stage, as well as 63 upregulated circRNAs and 70 downregulated circRNAs during the immature to the mature B-cell stage. Overall, researchers identified 1005 circRNAs present in at least one of the four above-mentioned B-cell subsets in the mouse bone marrow cells; this points to tight and precise regulation of B-cell development by circRNAs (accompanied by miRNA/mRNA network). Moreover, they discovered novel_circ_000317 and novel_circ_000383 and identified them as sponges of mmu-miR-15a-3p and mmu-miR-3059-5p, which target the gene Lair1 in the pro-B to pre-B cell transitional stage [69].

### 3.2. Role of circRNAs in DLBCL

CircRNAs have both diagnostic and prognostic value when used as biomarkers in patients with DLBCL. A number of studies have identified specific circRNAs that have been implicated in the development and progression of DLBCL, including halting the development of DLBCL. Hu Y et al. (2019) [70] first evaluated the characteristics of circRNA in DLBCL, focusing on circAPC, a back-spliced product from exon 7 to exon 14 of the *APC* gene. They found that circAPC acted as a sponge for miR-888, and by countering the repressive effects of miR-888 there was resulting upregulation of APC. In addition, circAPC was able to bind to TET1, a DNA demethylase enzyme that upregulates *APC* mRNA expression, resulting in increased transcription of APC. The authors concluded that circAPC was downregulated in tissue samples from DLBCL patients, and when circAPC was added to samples in vitro and in vivo, DLBCL proliferation was suppressed. Another circRNA, circEAF2, was also found to influence APC in Epstein–Barr virus (EBV) positive DLBCL patients, where it impeded cancer progression by stimulating apoptosis and sensitizing lymphoma cells to epirubicin, an anthracycline medication [71]. The authors proposed that EBV infection in DLBCL might alter RNA splicing, which accounts for the downregulated circEAF2 expression. Interestingly, there was no change to the expression of linear EAF2 in patient samples, indicating that the effect was on RNA splicing. A recent study by Zhou J et al. (2023) [72] found another downregulated circRNA in EBV+ DLBCL, circSPEF2 (hsa_circ0128899). CircSPEF2 was found to inhibit the proliferation of DLBCL cells and enhanced apoptosis through the expression of pro-apoptotic proteins Bax and Caspase-3. CircSPEF2 was also found to target miR-16-5p, a potential carcinogenic factor identified in multiple cancers. The researchers found that miR-16-5p increased the proportion of Treg cells and decreased the amount of CD4 cells in culture. A direct relationship was demonstrated between *BACH2* (BTB and CNC homolog 2, B cell specific transcription factor) and circSPEF2, such that silencing *BACH2* expression reversed the inhibitory effects of circSPEF2 on DLBCL cell proliferation and apoptosis. Through the miR-16-5p/*BACH2* axis, circSPEF2 was able to hinder the development of DLBCL [68]. In general, *BACH2* works as tumor suppressor (in pre-B cells) through MYC inhibition. Ichikawa S et al. (2014) described the important clinical role of *BACH2* in the prognosis of DLBCL. Concretely, that increased expression of *BACH2* correlates with worse prognosis and shorter OS of DLBCL patients [73]. The prognostic value of circRNAs is further illustrated by recently identified hsa_circ_0007099, which sponges 6 miRNAs (miR-188-3p, miR-1256, miR-1184, miR-338-3p, miR-495-3p, miR-495-5p), and results in the over-expression of *PIP4K2A* (phosphatidylinositol-5-phosphate 4-kinase type 2 lipid kinase A). PIP4K2A regulates cell proliferation, differentiation, and motility, and is associated with adverse OS of DLBCL patients [74].

CircRNAs are not only associated with the suppression and slowing of DLBCL, unfortunately, but also its progression. A study by Liu W et al. (2021) [75] found that circ_OTUD7A also acted as a miRNA sponge, but was able to promote the progression of DLBCL via the miR-431-5p/*FOXP1* pathway, implicating it as a potential oncogene. Lin et al. [76] identified circ_0003645 in DLBCL tissue samples and showed that circ_0003645 increased both the proliferation of cancer cells and glycolysis via the miR-335-5/NFIB pathway. Circ_0003645 positively regulated *NFIB* expression, which itself is targeted by miR-335-5p and was negatively correlated with miR-335-5p expression in DLBCL samples. This feature of circ_0003645 makes it a potential target for future DLBCL therapy. Another example of circRNA which promotes DLBCL progression is circCFL1. Authors revealed that circCFL1 sponges miR-107, which stimulates HMGB1 and thus cell proliferation [77].

Even with treatment by the current first-line therapy (R-CHOP), 40% of patients develop resistance and have relapsed/refractory disease [78]. Dong L et al. (2022) [79] identified circPCBP2 in DLBCL patient samples, and showed that it sponges miR-33a/b, a potential tumor suppressor that acts by suppressing the stemness, or ability to self-differentiate, proliferate, and renew [80]. CircPCBP2 knockdown, or conversely miR-33a/b overexpression, improved the pro-apoptotic effects of CHOP therapy in DLBCL cells [79].

It has recently been demonstrated that total cell-free RNA concentrations increase in DLBCL patients, but levels decreased after complete and successful R-CHOP therapy [81]. Together, these findings reinforce the involvement of circRNA (among other cfRNA = cell free) in patient’s resistance to therapy and suggest circPCBP2 as another therapeutic target to improve the effectiveness of R-CHOP therapy in DLBCL patients.

A recent study found that *PAX5* acts as a B-cell-specific activator, binding to the *GINS1* promoter region [82]. Moreover, the circRNA product of back splicing of *PAX5* pre-mRNA, circ1857, was able to promote *GINS1*, resulting in increased DNA replication in a coordinated way. To our knowledge, this is the first study to show this coordinated pattern between circRNA and the parental mRNA product, where both act on the same target. The study also indicated *PAX5* and circ1857 as potential oncogenes in some DLBCL.

CircRNAs could promote tumorigenesis/lymphomagenesis by interaction with proto-oncogenes, such as *MYC*. A study by Yang Q et al. (2017) [83] found that circAmotl1 (hsa_circ_0004214; from angiomotin like1 gene) was significantly upregulated (by circRNAs-seq) in cancerous cell lines in comparison to the controls; and possesses proliferative potential by interaction with c-myc. The authors showed that ectopic expression of circAmotl1 stabilizes c-myc and upregulates its targets, thus increasing binding affinity of c-myc to many promoters (HIF-1α, Cdc25a, ELK-1, JUN) and contributes to the tumorigenesis/lymphomagenesis. In addition, circ-Amotl1 co-localizes with c-myc in the cell nucleus and thus prevents c-myc degradation [83]. The brief summary about the role of circRNAs in DLBCL mentioned in the text above shows the Table 2.

In brief, the relationship between circRNA and miRNA appears to play an important role in the development of DLBCL and in the effectiveness of therapies.

## 4. Interaction and Regulation of circRNAs/miRNAs (Sponging)

**Figure 8:**
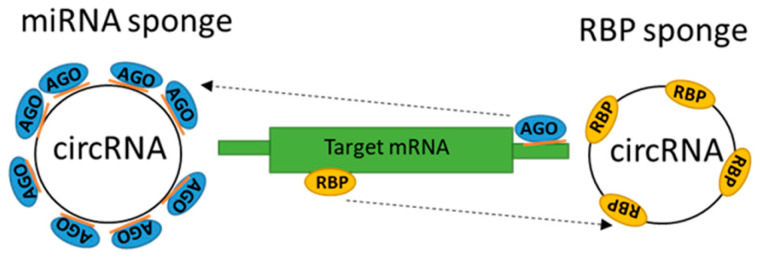
Schematic diagram of circRNA functioning as miRNA/RBP sponge. CircRNA contains several conserved miRNA target sites that can bind miRNAs, facilitate a specific miR-AGO interaction, and thus suppress concrete miRNA activity in many diseases (leukemia, lymphoma, hepatocellular carcinoma, colorectal cancer, diabetes, and others). AGO = Argonaute protein; RBP = RNA-binding protein.

In the previous chapter, we described how circRNAs regulate miRNAs expression, but how this mechanism works and is regulated is the focus of this chapter. In general, circRNAs, along with many other functions (see Figure 7), also regulate miRNA expression by (1) sponging (miRNA sponge, inhibition), (2) modulating the transcription factors which are a part of miRNA biogenesis, or (3) by sponging the set of miRNAs/RBPs involved in the regulation of factors during miRNA biogenesis [84]. The general scheme of interaction of circRNAs with miRNAs as a miRNA sponge or RBP sponge is depicted in Figure 8.

The subcellular localization of circRNAs determines their concrete function. For example, in the cytoplasm circRNAs sponge miRNAs but nuclear circRNAs act primarily as transcriptional regulators through binding proteins [70].

**Figure 9:**
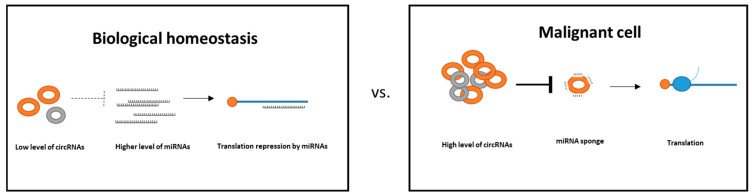
Summary scheme of circRNA and miRNA interaction in homeostasis vs. in malignant cells. In the case of homeostasis, cells express a low level of circRNAs with a high level of miRNAs that results in the translation repression of targeted mRNAs. In the case of malignancy, levels of circRNA increase and inhibit expression of miRNAs by sponging, which results in increased expression of oncogenes.

MiRNA sponge mechanisms use complementarity with activated miRNAs. The healthy cells express circRNA in very modest levels in comparison to elevated levels in malignant cells. In the case of balanced expression of miRNAs, translation of oncogenic genes is suppressed to maintain biological homeostasis. Deregulated expression of circRNA in malignant cells inhibits miRNA activity. Therefore, in malignant cells, due to disturbed biological homeostasis, an imbalance in the circRNA-miRNA axis causes upregulation of oncogenic targets (Figure 9) [85].

In the mechanism of miRNAs’ sponging by circRNAs, the number of binding sites on circRNAs seems to be an important parameter of inhibition efficacy/intensity. Some circRNAs are rich in miRNA-binding sites and can act as highly effective ceRNA (competing endogenous RNA) in cells, where they compete for binding sites on miRNAs and thus facilitate tumorigenesis [86]. However, most circRNAs have only one or a couple of binding sites for miRNAs. Interestingly, some circRNAs do not need miRNA binding, for example, in a recent study the authors found that circPVT1 in B-cell lymphomas acts independently of miRNAs binding (they do not explain the exact mechanism of action). Additionally, the locus for this circRNA is very close to the *MYC* gene locus, which could explain the implication of circPVT1 in cell proliferation [87].

CircRNAs compete with miRNAs (binding sites) and indirectly regulate the translation of targeted mRNAs. In other words, there exists a circRNA/miRNA/mRNA network. In general, the binding of tumor-suppressive miRNAs to circRNAs transcripts unblocks oncogenic miRNA targets by miRNA-dependent inhibition. Recently, researchers discovered the novel mechanism of RNA circularization associated with miRNA sponge formation (sponging), concretely by binding CUUCC pentanucleotide motifs on circRNA. Importantly, researchers confirmed that RNA circularization is miRNA-dependent. Their data show that synthesis of circRNA hsa-PHF2_0015 is induced by specific and precise binding of miR-7 to CDR1as mRNA and leads to the formation of a triple helix, which stabilizes the interactions of circRNA/miRNA/mRNA [88]. Furthermore, to check this mechanism on additional circRNAs, researchers analyzed over 341,000 human circRNAs deposited in the recently published CircAtlas database [89] and found out that plenty of circRNAs are rich in miRNA seed/binding sequences (1476 overall and 46 human circRNAs containing at least 20 and 50 pentanucleotide repeats = binding sites) [88].

Another sponging mechanism of circRNAs works through RNA binding proteins (RBPs) as described in Figure 8. This is performed through their sequence-specific and structural specific motif; moreover, circRNAs are able to store, sort, and localize RBPs [90]. An example of a circRNA and RBP interaction is between circFoxo3 (Forkhead box O transcription factor) and specific proteins that regulate cell cycle progression. Overexpression of circFoxo3 in non-cancer cells (healthy cells) results in cell cycle progression [91]. In contrast, when circFoxo3 is silenced, the cell cycle becomes suppressed through interactions with cell cycle proteins cyclin-dependent kinase 2 (CDK2) and cyclin-dependent kinase inhibitor 1 (or p21) [91]. The FOXO family may be aberrantly expressed or mutated in malignancies where they work as tumor suppressors by inhibiting cell growth, inducing apoptosis of cells, and by acting as mediators of cell homeostasis (reviewed in Hornsveld M et al. 2018 [92,93]). In DLBCL, *FOXO3a* behaves as a tumor suppressor when, after acquisition of ibrutinib resistance, the leukemic cells do not undergo programed cell death. Researchers created ibrutinib resistant cells (RIVA, MEC-1 cell lines), where detected upregulation of pFOXO3aSer253 resulted in its decreased accumulation in the nucleus and transcriptional inhibition of pro-apoptotic proteins PTEN and BIM [94]. Interestingly, *FOXO3* is a direct target of miR-155, so high expression levels of miR-155 reduces the level of *FOXO3* mRNA in DLBCL [95]. Inhibition of miR-155 induces apoptosis, reduces cell proliferation (in addition, our data support this notion Golovina E et al. 2024 [95]), and increases the expression of *FOXO3* mRNA [96].

A biophysical study revealed that the abovementioned sponging mechanisms of circRNA are efficient, and this leads to miRNA suppression through interactions with binding sites (the more the better), even with low concentrations of circRNAs in the cell (e.g., similar efficacy of inhibition was observed with 200 circRNAs and 12,000 transcription factors (TF)). Additionally, circRNAs remove (decoy) miRNAs faster, more efficiently, but less precisely in comparison to the classic TF-dependent regulatory loop [97]. The efficacy of this miRNA inhibition/sponging mediated by circRNA is shown on a number of inhibited miRNA molecules. Interestingly, the large internal noise (the stochasticity of biochemical interaction of particles) from multiple binding sites (seven) in circRNAs represents an important mark of this regulatory loop [97]. Duk and Samsonova generated an external noise vector to suppress the internal noise to examine the effect of internal noise. They found that the miRNA suppression was more efficient in the circRNA-dependent regulatory loop containing more (seven) binding sites in comparison to one binding site [97]. Thus, the number of binding sites in circRNAs is a key factor for effective miRNA sponging but dependent on saturation of miRNA binding sites. An example of a circRNA with multiple binding sites is ciRS-7, which has 73 conserved miRNA binding sites [98]. CiRS-7 is resistant to miRNA-mediated target destabilization and strongly inhibits miR-7 expression, which leads to an increase in miR-7 targets [98]. The summary Table 3 below includes list of circRNA and corresponding miRNA regulated by sponging in DLBCL.

Taken together, current research shows that circRNAs are efficient regulators of mRNA expression and act through sponging of miRNAs, which is also dependent on the number of binding sites of miRNAs on circRNAs and on miRNA saturation in the cell. Because circRNAs are reliable regulators of mRNA expression, this mechanism could be used in gene-targeted therapy, not only for DLBCL.

### Potential Reasons of Deregulation of circRNAs in the Malignant Cells

As deregulation of circRNAs in the circRNA/miRNA/mRNA network is usually a feature of malignancy/leukemia, we do not know the exact causes of the upregulation of circRNA in malignant cells. We could hypothesize that one of the reasons could be some mutation in the spliceosome genes such as *SF3B1*, *SRSF2*, and *U2AF1* (these genes are often mutated in malignant cells) as they regulate biogenesis of circRNAs [101]. Next, circRNA translation is positively regulated by internal ribosome entry site-like elements [102], thus, these could also be one of the potential enhancers. Other regulatory molecules of circRNA biogenesis are RNA helicases that promote the unwinding of RNA during splicing and translation [102]. Biogenesis and function of circRNA is modulated by m6A modification, concretely this modulation results in inhibition of innate immunity by blocking RIG-I activation [103]. RNA-Binding Proteins also act on splicing and are thus potential regulators of circRNA production [102]. Another possible reason of upregulation of circRNAs in the malignant cells could be the mutation of trans-acting factors, such as quaking (QKI). By interacting with pre-mRNA and bringing circ-forming exons closer together, QKI might stimulate the biogenesis of circRNA [104]. Cis-regulatory elements also act on the circRNA production and could therefore be implicated in the aberrant expression of circRNAs [102]. There is also evidence that *ADAR1*, *MBNL1,* and *QKI* are involved in the process of RNA circularization [105].

However, there is still no direct evidence that the spliceosomes, quaking, RNA helicases, RNA binding proteins, and other abovementioned factors initiate leukemia/cancer development by affecting the production or biogenesis of circRNA, thus, it could be a direction of continued research and exploration in the future.

## 5. The Role of circRNAs in Pathways

As described in the above-mentioned chapters, circRNAs indirectly regulate many biological pathways in both normal and leukemic cells. This chapter will discuss the key pathways related to B-cells and lymphomas.

### 5.1. NF-κB Pathway

NF-κB (Nuclear Factor kappa-light-chain-enhancer of activated B-cells) represents a prototypical proinflammatory signaling pathway, which is associated with the activation of proinflammatory cytokines, such as interleukin 1 (IL-1) and tumor necrosis factor α (TNFα), and expression of molecules that control the transcription of DNA, cytokine production, cell survival, chemokines, and adhesion molecules [106]. The activation of the NF-kB pathway is more frequently observed in the ABC-DLBCL subtype [107].

Unfortunately, today there are no known circRNAs that regulate the NF-kB pathway in DLBCL; however, it is known that NF-kB activity is regulated by miRNAs. Specifically, miR-155 and miR-146a together are involved in the regulation of the intensity and duration of the inflammation response. NF-kB activity is controlled by miR-155 and miR-146a in two steps. First, NF-kB stimulates expression of miR-155 that targets *SHIP1*, which then activates the IKK signalosome complex in a PI3K/Akt-dependent manner, creating a positive feedback loop necessary for signal enhancement. Second, NF-kB slowly stimulates the expression of miR-146a and, in this case, forms a negative feedback loop by targeting *IRAK1* and *TRAF6*, which results in the attenuation of NF-kB activity in later phases of inflammation. Such common capabilities of miR-155/miR-146a/NF-kB regulatory loops leads to the optimal activity of NF-kB signaling after inflammatory stimuli [108].

Recent work by Carreras J et al. (2024) described the important prognostic role of NF-ĸB subunit—the *RELB* proto-oncogene. Authors find out that overexpression of *RELB* is associated with a favorable prognosis for DLBCL patients [109].

As miRNAs are regulated by circRNAs, it is only a matter of time until novel circRNAs with regulatory activity in the NF-kB pathway in DLBCL are discovered.

### 5.2. PI3K/AKT Pathway

The PI3K/AKT pathway is important for B-cell development [110]. It has been described that exogenous overexpression of CircCFL1 (by plasmid transfection) in DLBCL cell lines OCI-Ly7 and OCI-Ly3 resulted in the upregulation of p-AKT, thus, CircCFL1 activated the AKT pathway and promoted the proliferation of DLBCL cells. The authors further found that CircCFL1 directly binds to miR-107 and, in doing so, CircCFL1 sponged miR-107. Overexpression of CircCFL1 led to tumor growth in mice xenograft model of DLBCL [77]. Another study demonstrated that downregulation of Circ_0000877 in DLBCL leads to inhibition of cell proliferation, glycolysis, and increased apoptosis. Circ_0000877 sponges miR-671-5p, as it contains binding sites for miR-671-5p [99]. Overexpression of miR-671-5p might diminish DLBCL development by decreasing cell proliferation and invasion [111].

Few described circRNAs regulate DLBCL development and proliferation. The number of known circRNAs involved in the PI3K/AKT pathway is expected to increase with time.

### 5.3. MYC Pathway

The *MYC* family contains several members, among them oncogenes such as *C-MYC*, *L-MYC*, and *N-MYC*, which regulate cell proliferation, apoptosis, DNA damage, cell cycle and if deregulated they stimulate leukemia/cancer development. Among *MYC* family members, *C-MYC* is the most studied due to its multifunctionality and multiple regulatory levels.

It was described that circAmotl1 stimulates nuclear translocation of *C-MY*C, upregulates *C-MYC* targets, and thus is indirectly involved in lymphomagenesis [83]. Because *C-MYC* mRNA is frequently upregulated in DLBCL [112] and its expression is regulated by circRNAs (circAmotl1), new possibilities arise for circRNAs to serve as diagnostic and therapeutic molecules. Chromosomal translocations are frequent in DLBCL and researchers have found that they could stimulate fusion-circRNAs (f-circRNA, created by fusion of two translocated genes) and initiate tumor formation [113].

As *MYC* is often overexpressed or mutated in DLBCL, a study of circRNAs that regulate *MYC* expression is of importance in further research.

### 5.4. Wnt Pathway

The name Wnt pathway comes from the portmanteau of gene Int1 (mouse proto-oncogene integration 1) and Wingless (it is the gene Int1in *D. melanogaster*) and was discovered by studying the oncogenic potential of retroviruses in breast cancer. This pathway, in general, regulates cell fate specification, cell proliferation, and cell migration. We recognize three pathways: (1) the canonical Wnt pathway, (2) the non-canonical planar cell polarity pathway, and (3) the non-canonical Wnt/calcium pathway [114].

A study by Hu Y et al. (2019) [70] found that circAPC is significantly downregulated in DLBCL and is negatively associated with the aggressiveness of DLBCL. CircAPC stimulates the expression of the host gene adenomatous polyposis coli (APC), which inactivates canonical Wnt/β-catenin signaling, and hampers growth of DLBCL cells [70]. A similar study by Li F et al. (2015) [100] described that circITCH (acting as tumor suppressor) suppresses the Wnt pathway indirectly through the inhibition of miR-7, miR-17, and miR-214 [100].

In summary, several circRNAs regulate the Wnt signaling pathway. There are still many unknown circRNAs whose role in leukemia is yet to be elucidated.

## 6. Visions and Prognosis for circRNA as Therapeutic Molecules

CircRNAs are often highly dysregulated, showing tissue or cell specific upregulation in different diseases. Therefore, they may provide an accurate indicator of disease development and progression, thus making them promising therapeutic molecules or targets. Targeted inhibition of circRNAs has greater therapeutic benefits and potential than targeted inhibition of a single miRNA or gene because circRNAs contain multiple miRNA binding sites. The biggest advantage of circRNAs lies in their circular structure, which provides consistency to their delivery and stability [115]. A disadvantage of circRNAs as biomarkers is the fact that the isolation of circRNAs is still relatively complicated, but this could be solved as detection techniques continue to improve.

Nowadays, there are many available techniques for how to modify genetic material in the cell, i.e., by CRISPR/Cas9/Cas13 (KO, KI), viral vectors (KO, KI), shRNA/siRNA (KO), nanoparticles or exosome delivery of circRNA molecules [116]. RNA interference (RNAi) is one of the most frequent methods used to assess the function of circRNA through a loss-of-function approach. Transcripts of circRNAs might be packed into viral vectors or oligonucleotide and then introduced to the target cells to mediate their therapeutic effects. CircRNA-based therapeutic approaches still need to go through several obstacles, such as off-target silencing via a miRNA-like effect [117]. When compared to RNAi, the CRISPR/Cas13 tool demonstrated low mismatch tolerance and more efficient knockdown of circRNAs [118]. In order to obtain high tissue/cell specificity, it is best to use nanoparticles [119]. To conclude, CRISPR/Cas13 seems to be most promising in the case of KO of some circRNAs, as it shows high specificity, efficiency, and provides the possibility of performing experiments in vivo. Considerable disadvantages of CRISPR are hidden side effects. Surprisingly, currently the most feasible approach is siRNA technology, thanks to its efficiency, specificity, and relatively low toxicity. As the circRNA field is relatively new, there are only a few clinical trials/studies with circRNAs as medicaments for DLBCL. As previously mentioned, a study on the role of circAPC as an efficient sponge of miR-888 was the first study to confirm circRNA as a biomarker. The authors further tested xenograft tumor mice models to determine if circAPC could also reduce the proliferation of cells in vivo. They successfully confirmed an antiproliferative effect of exogenous overexpression of circAPC in mice, demonstrating tumor reduction. To assess the clinical application of circAPC, the authors used DLBCL patient tissue and plasma samples. The expression level (qRT-PCR) of circAPC in tissue and plasma of DLBCL samples was significantly low, as they had hypothesized, suggesting that the measurement of circAPC expression could be a useful indicator of DLBCL diagnosis and prognosis [70]. Additionally, APC is a key inhibitor of the canonical Wnt/β-catenin pathway, stimulates phosphorylation and subsequent proteolytic degradation of β-catenin, and by this reduces the accumulation of β-catenin in the nucleus [120]. Another study investigated the role of the circPCBP2/miR-33a/b/PD-L1 axis in DLBCL development and sensitivity to chemotherapy. The expression level of circPCBP2 was increased in tissue samples of DLBCL patients and positively correlated with survival rate. To examine the effect of circPCBP2 on cancer progression, the authors abolished its expression through sh-circPCBP2. In addition to cancer cell progression, they also investigated the stemness of cancer cells by measuring pluripotent transcription factors (OCT4, Nanog, SOX2, ALDH1A1). The inhibition of circPCBP2 by sh-circPCBP2 downregulates expression of abovementioned pluripotent transcription factors, so stemness is reduced. Interestingly, sensitivity to CHOP therapy increased. Using RNA-RIP methods, the authors revealed that circPCBP2 directly binds to miR-33a/b, and miR-33a was associated with a response to R-CHOP therapy in DLBCL [121]. As PD-L1 was already associated with the stemness of cancer cells, the authors measured the expression level of PD-L1 in DLBCL cells and found that sh-circPCBP2 significantly reduced *PD-L1* mRNA levels in DLBCL cells. They also investigated the relationship between miR-33a/b and PD-L1 by detecting mRNA levels of PD-L1 in DLBCL tissues and cells. As expected, PD-L1 mRNA was increased, and miR-33a/b levels were decreased in human DLBCL tissues. Bioinformatics analysis (starBase) revealed a potential binding site for miR-33a/b on PD-L1 mRNA, thus miR-33a/b might target PD-L1 mRNA [70]. The gene *HMGB1* (high mobility group box 1) is associated with malignancy, cell proliferation, inflammation, and metastasis [122]. A study conducted by Chen et al., 2020 aimed to investigate the effect of circCFL1 on the malignant progression of DLBCL. To do this, researchers used an expression plasmid to overexpress circCFL1 and siRNA to knock down the expression of circCFL1 in DLBCL cell lines. Through RNA-pull experiments, they confirmed direct binding of miR-107 with HMGB1. Using the dual-luciferase reporter gene system, the authors demonstrated that circCFL1 targets miR-107. This is the first study to associate circRNA with the proliferation of DLBCL cells. Moreover, they discovered that circCFL1 can increase *HMGB1* expression by targeting miR-107, which activates the HMGB1 signaling pathway and raises p-AKT, p-ERK, and p-STAT3 phosphorylation levels [77].

While we provide all current knowledge about the implication of circRNAs in DLBCL here, it also highlights the limited number of available studies and the need for further investigation.

To conclude, circRNAs’ expression is tissue-specific and dynamic (especially during chemotherapy), but measuring circRNA during disease progression could provide a real-time indication of the state of the disease and help to determine the effectiveness of treatment. Based on recent research, circRNAs primarily control cell activity and tumor growth by sponging to miRNAs and RBPs. The study of the circRNA/miRNA axis is substantially aided by the advancement of bioinformatics technologies. Therefore, as we gain more knowledge about circRNAs and their role in lymphomas, the closer we will be to efficient diagnosis and therapy of DLBCL.

## 7. Conclusions

DLBCL represents a very aggressive type of B-cell lymphoma, with 30–40% of patients experiencing no benefit from first-line therapy or experiencing relapse, despite multiple therapeutic options. In addition, early detection of DLBCL is still problematic. CircRNAs are relatively novel regulatory molecules that could be used as biomarkers or therapeutic modalities. The main, and most studied, action of circRNAs is competing for binding sites with miRNAs, termed sponging. CircRNAs negatively regulate miRNA expression and are stronger predictors of early disease prognosis when compared to miRNAs. CircRNAs are involved in the regulation of cellular pathways. Detailed knowledge about the circRNA/miRNA/mRNA regulatory network in DLBCL cells opens new possibilities for more specific and efficient therapeutic medicaments in DLBCL.

## Figures and Tables

**Table 1:** Summary of miRNAs detected in DLBCL.

miRNA	Function/Role	Pathway	Sample Origin	References
miR-15a, miR-16-1, miR-21, miR-29c, miR-155	Prognosis/Progression	MYC, PI3K	Serum	[34]
miR-200c-3p, miR-421 and miR-324-5p	Progression/Therapy	MYC, NFkB	Serum	[36]
miR-34a, miR-27b, miR-21, miR-22	Diagnosis/Prognosis	MYC, NFkB, PI3K	PBMC	[35]
miR-181, miR-195, miR-26a, miR-101	Cell cycle	MYC, NFkB	Lymph nodes	[35]
miR-497, miR-199a miR-130a, miR-125b	Chemoresistance	MYC, NFkB	Tumor/Tissue	[35]
miR-155-5p and miR-221-3p	Diagnosis	MYC	FFPE	[37]
miR-125b, miR-155	Prognosis	MYC, NFkB	FFPE	[38]
miR-129-2-3p, miR-4464, miR-3150b-3p, miR-138-5p, miR-129-5p, miR-511-5p, miR-205-5p, miR-3652	Diagnosis/Prognosis	TP53, MYC	FFPE	[39]
miR-146a	Prognosis/Therapy	PI3K/AKT	Serum	[40]

**Table 2:** Summary of circRNAs detected in DLBCL.

circRNA	Function/Role	Sample Origin	References
circAPC	Sponges miR-888	FFPE	[70]
circEAF2	Apoptosis of cells	FFPE	[71]
circSPEF2	Apoptosis of cells	Tissue	[72]
hsa_circ_0007099	Prognosis	Tissue	[74]
circOTUD7A	Sponges miR-432-5p, disease progression	Tissue	[75]
circ0003645	Proliferation of cells	FFPE	[76]
circCFL1	Diseases progression	DLBCL cell lines	[77]
circPCBP2	Sponges miR-33a/b	Tissue	[79]
circ1857	Oncogene	Tissue	[82]
circAmotl1 (hsa_circ0004214)	Tumorigenesis/Lyphomagenesis	Cancer cell lines	[83]

**Table 3:** Summary table of circRNA and corresponding miRNA regulated by sponging in DLBCL.

circRNA	miRNA Sponge	References
circAPC	miR-888	[70]
circ0007099	miR-188-3p, miR-1256, miR-1184, miR-338-3p, miR-495-3p, miR-495-5p	[74]
circOTUD7A	miR-432-5p	[75]
circCFL1	miR-107	[77]
circPCBP2	miR-33a/b	[79]
circ0000877	miR-671-5p	[99]
circITCH	miR-7, miR-17, miR-214	[100]
